# Successful treatment of Premenstrual dysphoric disorder with irritable bowel syndrome using sulpiride

**DOI:** 10.1192/j.eurpsy.2024.1437

**Published:** 2024-08-27

**Authors:** A. A. Alageel

**Affiliations:** College of medicine, Imam Mohammad Ibn Saud Islamic University (IMSIU), Riyadh, Saudi Arabia

## Abstract

**Introduction:**

Premenstrual dysphoric disorder (PMDD) is prevalent, more severe than premenstrual syndrome(PMS), and a challenging disorder. The first line of treatment is pharmacotherapy. Non-pharmacological therapy includes aerobic exercise, consumption of complex carbohydrates and frequent meals, relaxation training, light therapy, sleep deprivation, and cognitive-behavioral therapy could be helpful

**Objectives:**

To our knowledge, there have not yet been any studies on this treatment option for PMDD with IBS

**Methods:**

a case report

**Results:**

A lady suffering from PMDD and irritable bowel syndrome (IBS) did not respond to antidepressants, painkillers, and melatonin. She used to sit at home and in her room these days, waiting for the PMDD severity to decrease. Her condition reached remission after taking a small dosage of sulpiride and stopped on the last day of the period. The patient is satisfied with the result since concerns about antidepressants are addressed and avoided. This case provides a new approach to using low-dosage sulpiride temporarily every month in patients with both PMDD and IBS

**Conclusions:**

Premenstrual dysphoric disorder is a challenging condition. The symptoms of PMDD are not continuous, and somatic symptoms are a significant component of both the diagnosis and the patient’s suffering. Choosing a suitable medication based on pros and cons contributes to successful treatment and patient satisfaction. This case provides a new approach to using low-dosage sulpiride in patients with both PMDD and IBS, but more studies are needed to confirm its efficacy and safety.

**Disclosure of Interest:**

None Declared

